# Impact of age and comorbidities on health-related quality of life for patients with prostate cancer: evaluation before a curative treatment

**DOI:** 10.1186/1471-2407-9-296

**Published:** 2009-08-24

**Authors:** Michael Pinkawa, Karin Fischedick, Bernd Gagel, Marc D Piroth, Branka Asadpour, Jens Klotz, Holger Borchers, Gerhard Jakse, Michael J Eble

**Affiliations:** 1 Department of Radiation Oncology, RWTH Aachen University, Pauwelsstrasse 30, 52057 Aachen, Germany; 2 Department of Urology, RWTH Aachen University, Pauwelsstrasse 30, 52057 Aachen, Germany

## Abstract

**Background:**

Interpretation of comparative health-related quality of life (HRQOL) studies following different prostate cancer treatments is often difficult due to differing patient ages. Furthermore, age-related changes can hardly be discriminated from therapy-related changes. The evaluation of age-and comorbidity-related changes was in focus of this study.

**Methods:**

HRQOL of 528 prostate cancer patients was analysed using a validated questionnaire (Expanded Prostate Cancer Index Composite) before a curative treatment. Patients were divided into age groups ≤65, 66-70, 71-75 and >75 years. The impact of specific comorbidities and the Charlson Comorbidity Index (CCI) were evaluated. The questionnaire comprises 50 items concerning the urinary, bowel, sexual and hormonal domains for function and bother. For assessment of sexual and hormonal domains, only patients without prior hormonal treatment were included (n = 336).

**Results:**

Urinary incontinence was observed increasingly with higher age (mean function scores of 92/88/85/87 for patients ≤65, 66-70, 71-75 and >75 years) - complete urinary control in 78%/72%/64%/58% (p < 0.01). Sexual function scores decreased particularly (48/43/35/30), with erections sufficient for intercourse in 68%/50%/36%/32% (p < 0.01) - a decrease of more than a third comparing patients ≤65 vs. 66-70 (36%) and 66-70 vs. 71-75 years (39%). The percentage of patients with comorbidities was lowest in the youngest group (48% vs. 66%/68%/63% for ages 66-70/71-75/>75 years; p < 0.05). A multivariate analysis revealed an independent influence of both age and comorbidities on urinary incontinence, specifically diabetes on urinary bother, and both age and diabetes on sexual function/bother. Rectal domain scores were not significantly influenced by age or comorbidities. A CCI>5 particularly predisposed for lower urinary and sexual HRQOL scores.

**Conclusion:**

Urinary continence and sexual function are the crucial HRQOL domains with age-related and independently comorbidity-related decreasing scores. The results need to be considered for the interpretation of comparative studies or longitudinal changes after a curative treatment.

## Background

The number of men diagnosed with localized prostate cancer has increased substantially since the advent of prostate-specific antigen (PSA) testing [1]. The incidence rates stabilized in the last years [2]. Only one randomized study of radical prostatectomy has shown that treatment reduces prostate cancer mortality compared to watchful waiting [3]. Alternatively, external beam irradiation or interstitial brachytherapy is offered frequently [4-8]. Despite advances in the primary treatments, no randomized controlled trial has proven the superiority of one modality in terms of cancer control. In the absence of mortality data, health-related quality of life (HRQOL) evaluations become important outcomes.

Commonly used grading systems are suitable to determine the incidence of most serious side effects, but not the precise significance of problems for HRQOL. Patients are subjectively assessing their problems to be of higher grades compared to the assessment obtained by the physician [9]. Many functions are already impaired before treatment. Important factors are the patient age and comorbidities. Only a minority of HRQOL studies in men with prostate cancer provide some form of comorbidity information [10].

Interpretation of comparative HRQOL studies following different prostate cancer treatments is often difficult due to differing patient ages. Patients receiving external beam radiation therapy are usually slightly older compared to patients receiving brachytherapy, and on average about ten years older compared to patients treated with radical prostatectomy [4-8]. Age-related changes can hardly be discriminated from therapy-related changes in these studies. Not only pre-treatment HRQOL, but additionally natural HRQOL changes can be expected to differ between these patient groups (e.g. changes of a 60 year old compared to a 70 year old patient in the subsequent ten years of life). The aim of this study was to evaluate age-related and comorbidity-related urinary, bowel, sexual and hormonal HRQOL differences of prostate cancer patients before a primary curative treatment.

## Methods

This study was based on 528 consecutive patients who were scheduled for any kind of a curative radiotherapy treatment (external beam irradiation, temporary or permanent brachytherapy) due to cT1-3N0M0 prostatic carcinoma in the years 2003-2007. All patients answered a validated questionnaire, the Expanded Prostate Cancer Index Composite (EPIC), before the treatment [11]. The questionnaire was handed over to the patients personally by one of the physicians. It comprises 50 items concerning the urinary, bowel, sexual and hormonal domains for function and bother. The multi-item scale scores were transformed lineary to a 0-100 scale, with higher scores representing better health-related quality of life. For a detailed analysis, questions in the urinary domain were classified into incontinence and irritation/obstruction subscales [11,12].

Data has been acquired in compliance with the Helsinki Declaration. The evaluation was approved by the local ethics committee. Informed consent was obtained from patients for the treatment. Patients were informed about the voluntary participation in the evaluation of quality of life data. An explicit statement about the voluntary character of the participation was heading every page of the questionnaire.

The EPIC was administered in German. It has been translated in the Department of Radiation Oncology and validated in the department of Medical Psychology and Medical Sociology of our institution [13]. Several studies have been already published [14-20]. These studies very well support the sensitivity of the questionnaire for quality of life changes after treatment in all domains. The relation between individual questions and quality of life scores in different domains as well the correlation of function and bother domains or changes with time have been elaborated in detail. In accordance with data published by Osoba et al. [21], mean HRQOL changes of below 5 points were defined as clinically not significant. For patients who indicated "a little" change, the mean change in scores was about 5 to 10, for "moderate" change, about 10 to 20; and for "very much" change, greater than 20. The EORTC (European Organization for Research and Treatment of Cancer) QLQ (Quality of Life Questionnaire)-C30, evaluated by Osoba et al., uses comparably to the EPIC a 0 to 100 scale. Though the assessment of a clinically significant threshold remains controversial, a 5-point threshold proved likewise to be clinically significant in our own experience [14-20].

The group has been divided into the age groups up to 65 (n = 116), 66-70 (n = 144), 71-75 (n = 158) und >75 years (n = 110) with the aim to discriminate purely age-related health-related quality of life (HRQOL) changes. Five-year increments have been estimated to be relevant intervals with an adequate patient number in each group to detect significant age-related differences. The Charlson Comorbidity Index (CCI) was calculated as a scale that considers both comorbidities and the patient age (starting at 50 years of age, each decade is counted as an extra point) [22].

Neoadjuvant hormonal therapy (NHT) was administered in 192 cases. In a preliminary analysis, mean HRQOL scores were found to be significantly lower in the sexual (function scores: -18 points) and hormonal (function scores: -11 points) domains. Therefore, all evaluations of sexual and hormonal symptoms and scores were limited to the group of patients without NHT (n = 336; i.e. prostate cancer not treated with any method before).

Statistical analysis was performed using the SPSS 14.0 (SPSS, Chicago, Ill), software. To explore statistical HRQOL score differences between different subgroups, the Mann-Whitney-U-test was used. Contingency table analysis with the chi-square test was performed to compare treatment groups with respect to categorical variables. In a logistic forward stepwise multivariate analysis, different risk factors were tested for their independence. For the evaluation of age and comorbidities, multivariate analysis was performed twice: 1. considering age and the presence of any comorbidity; 2. considering age and various specific comorbidities. As the CCI scale is considering both age and comorbidities, it cannot be considered in this multivariate analysis. Hazard ratios in dependence on the CCI scale were therefore reported separately. The global significance level for all statistical test procedures conducted was chosen as α = 5%. All statistical analyses were conducted in an explorative manner. Thus, with consideration of the explorative character of the analysis, p-values < 0.05 can be interpreted as statistically significant test results.

## Results

Patient characteristics (Table 1) demonstrate a well balanced distribution of a prior hormonal therapy over the different age groups. The prostate volume was slightly increasing with the patient age. The highest percentage of tumors with a PSA ≥10 ng/mL and a Gleason score ≥7 was found in the oldest age group. The percentage of patients with at least a single comorbidity was lowest in the youngest age group (significant difference in comparison to all other age groups) - four of the most frequent comorbidities are listed additionally in Table 1. The CCI demonstrates highly significant differences. Patients older than 70 years reached at least five points due to their age (three points) and the diagnosis of prostate cancer (two points).

**Table 1:** Patient characteristics (n = 528)

	≤65 years (n = 116)	66-70 years (n = 144)	71-75 years (n = 158)	>75 years (n = 110)	significant difference
patient age, years mean ± SD; median	62 ± 4; 63	68 ± 2; 68	73 ± 2; 73	78 ± 2; 77	all comparisons (p < 0.01)

prostate volume, cc mean ± SD; median	42 ± 20; 35	44 ± 20; 39	45 ± 23; 40	46 ± 21; 42	-

PSA<10 ng/mL, n (%)	66 (57)	93 (65)	95 (60)	55 (50)	66-70 vs. >75 years (p < 0.05)

biopsy Gleason score <7; n (%)	72 (62)	90 (63)	98 (62)	55 (50)	66-70 vs. >75 years (p < 0.05)

prior hormonal therapy; n (%)	46 (40)	50 (35)	55 (35)	41 (37)	-

comorbidities; n (%)	56 (48)	95 (66)	108 (68)	69 (63)	≤65 vs. 66-70/71-75/>75 years (p < 0.05)

hypertension; n (%)	24 (21)	44 (31)	53 (34)	27 (25)	≤65 vs. 71-75 years (p < 0.05)

coronary heart disease; n (%)	17 (15)	29 (20)	43 (27)	30 (27)	≤65 vs. 71-75/>75 years (p < 0.05)

diabetes; n (%)	13 (11)	16 (11)	17 (11)	16 (15)	-

COPD; n (%)	7 (6)	13 (9)	17 (11)	12 (11)	-

CCI<5/5/>5; n (%)	86/17/13 (74/15/11)	69/49/26 (48/34/18)	0/85/73 (0/54/46)	0/47/63 (0/43/57)	for all (p < 0.01), with exception 71-75 vs. >75

SD: standard deviation; PSA: prostate specific antigen; COPD: chronic obstructive pulmonary disease; CCI: Charlson Comotbidity Index

**Figure 1:**
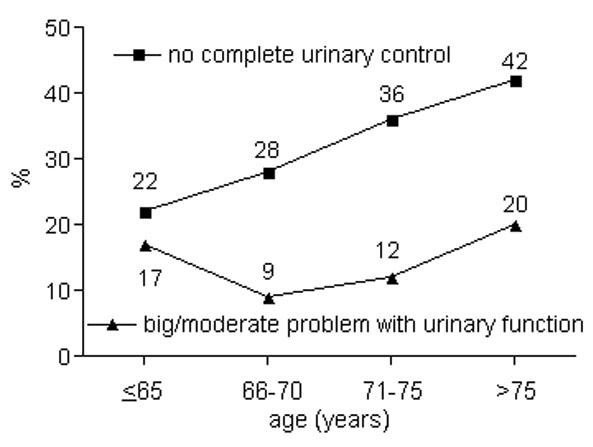
**Percentage of patients with no complete urinary control (p < 0.01) and big/moderate problem with urinary function (p = 0.04)**.

**Figure 2:**
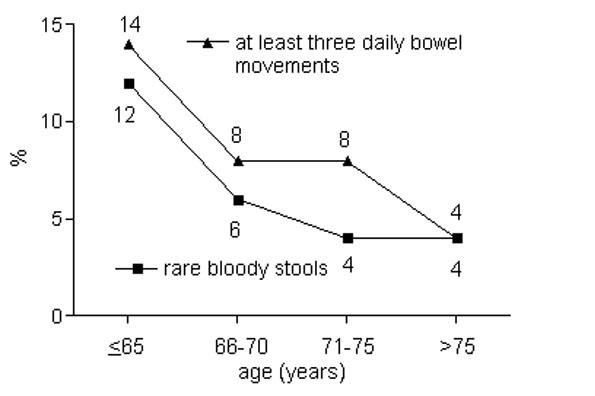
**Percentage of patients with at least three daily bowel movements (p = 0.04) and at least rare bloody stools (p = 0.02)**.

**Figure 3:**
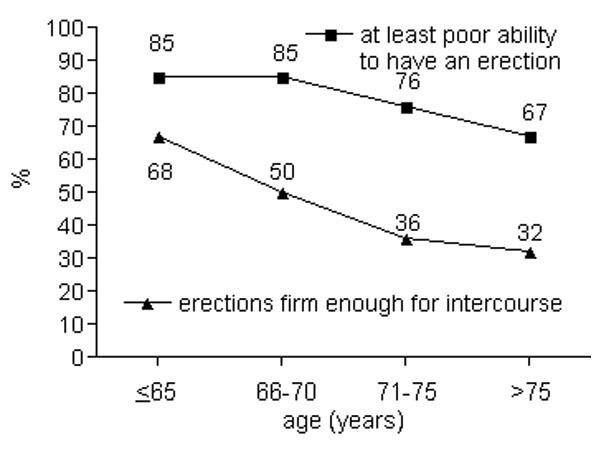
**Percentage of patients with at least poor ability to have an erection (p = 0.04) and erections firm enough for sexual intercourse (p < 0.01)**.

**Figure 4:**
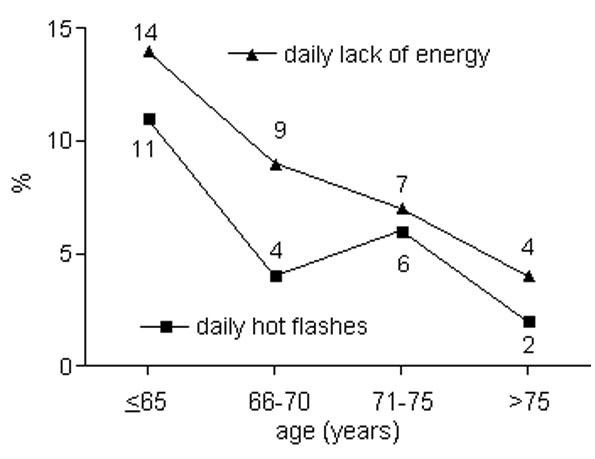
**Percentage of patients with daily lack of energy (p = 0.18) and daily hot flashes (p = 0.18)**.

Urinary incontinence was observed increasingly with higher age; urinary bother scores decreased. Sexual function scores fell most impressively, but sexual bother scores did not change as markedly (Table 2). The results of some individual questions demonstrating significant changes with increasing patient ages are presented in Figures 1, 2, 3 and 4 (only tendencies in the hormonal domain). Some ability to have an erection remains even in the oldest age group, but in only 32% firm enough for sexual intercourse. The percentage of patients with erections firm enough for sexual intercourse decreased more than a third comparing patients up to 65 vs. 66-70 years (36%) and 66-70 vs. 71-75 years (39%), respectively. Even clearer than the different age groups, a CCI>5 was particularly predisposing for significantly lower urinary function/bother (including both continence and irritation/obstruction) and lower sexual function/bother scores (Table 3).

**Table 2:** Function and bother scores (mean ± standard deviation; median) in dependence on age group

	≤65 years	66-70 years	71-75 years	>75 years
urinary function	94 ± 13; 100	92 ± 15; 100	91 ± 14; 100	92 ± 12; 94

continence function*	***92 ± 18; 100***	***88 ± 21; 100***	***85 ± 23; 100***	***87 ± 20; 94***

irritative/obstructive function	95 ± 13; 100	97 ± 12; 100	98 ± 10; 100	98 ± 9; 94

***urinary bother†***	***82 ± 20; 89***	***84 ± 20; 91***	***82 ± 18; 89***	***79 ± 19; 86***

continence bother	93 ± 22; 100	90 ± 25; 100	90 ± 25; 100	90 ± 22; 100

irritative/obstructive bother	80 ± 20; 90	83 ± 20; 90	81 ± 18; 85	79 ± 18; 83

bowel function	91 ± 11; 96	93 ± 9; 96	93 ± 9; 96	93 ± 9; 96

bowel bother	93 ± 14; 100	93 ± 14; 100	95 ± 10; 100	93 ± 13; 100

***sexual function***‡	***48 ± 25; 50***	***43 ± 23; 50***	***35 ± 23; 32***	***30 ± 24; 28***

sexual bother	68 ± 32; 75	64 ± 34; 75	56 ± 36; 56	60 ± 33; 63

***hormonal function*§**	***88 ± 17; 100***	***91 ± 16; 100***	***92 ± 13; 100***	***95 ± 10; 100***

hormonal bother	89 ± 16; 96	91 ± 15; 100	92 ± 14; 96	94 ± 11; 100

*significant difference ≤65 vs. 71-75/>75 years (p < 0.01)

† significant difference 66-70 vs. >75 years (p < 0.01)

‡ significant difference ≤65 vs. 71-75/>75 and 66-70 vs. 71-75/>75 years (p < 0.01)

§ significant difference ≤65 vs. >75 years (p = 0.03)

**Table 3:** Function and bother scores (mean ± standard deviation; median) in dependence on Charlson Comorbidity Index (CCI)

CCI	<5	5	>5
urinary function*	***94 ± 12; 100***	***93 ± 12; 100***	***89 ± 16; 94***

continence function*	***92 ± 17; 100***	***90 ± 18; 100***	***82 ± 25; 100***

irritative/obstructive function	97 ± 12; 100	97 ± 9; 100	96 ± 13; 100

urinary bother*	***85 ± 19; 93***	***84 ± 16; 89***	***77 ± 22; 82***

continence bother*	***93 ± 20; 100***	***93 ± 20; 100***	***86 ± 29; 100***

irritative/obstructive bother*	***84 ± 19; 90***	***83 ± 16; 85***	***76 ± 21; 80***

bowel function	93 ± 9; 96	93 ± 8; 96	91 ± 10; 96

bowel bother	94 ± 13; 100	94 ± 11; 100	92 ± 15; 100

sexual function*	***49 ± 22; 51***	***37 ± 24; 37***	***31 ± 24; 29***

***sexual bother***†	***68 ± 31; 75***	***64 ± 34; 69***	***52 ± 36; 50***

hormonal function	92 ± 14; 100	90 ± 13; 100	90 ± 13; 100

hormonal bother	92 ± 13; 100	90 ± 15; 96	90 ± 15; 96

*significant difference <5/5 vs. >5 (p < 0.01)

† significant difference <5 vs. >5 (p < 0.01) and 5 vs. >5 (p = 0.03)

The presence of even a single comorbidity was associated with decreased HRQOL scores (Table 4). A mean decrease of at least 5 points has not been observed in the rectal domain. Parallel with a higher patient age, decreasing scores associated with a comorbidity were found for the urinary continence function, urinary bother (specifically coronary heart disease and diabetes) and sexual function (strong impact of diabetes). A contrary effect resulted in the hormonal domain - decreasing scores associated with coronary heart disease and COPD, but increasing scores associated with age.

**Table 4:** Association of health-related quality of life score decrease with comorbidities (mean ± standard deviation; only significant differences of at least 5 points presented)

	comorbidity (n = 328/178*)	coronary heart disease (n = 119/63*)	diabetes (n = 62/37*)	COPD (n = 49/29*)
urinary function	-	-	-	-

continence function	5 ± 2	6 ± 2	7 ± 3	-

irritative/obstructive function	-	-	-	-

urinary bother	6 ± 2†	7 ± 2†	9 ± 3†	-

continence bother	-	-	-	-

irritative/obstructive bother	5 ± 2†	6 ± 2†	8 ± 3†	-

sexual function	9 ± 3†	-	16 ± 4†	-

sexual bother	10 ± 4	-	18 ± 6†	-

hormonal function	5 ± 2†	7 ± 2†	-	5 ± 3†

hormonal bother	5 ± 2†	6 ± 2†	-	5 ± 3†

COPD: chronic obstructive pulmonary disease

*patients without a prior hormonal therapy (analysis of sexual and hormonal domains)

† p < 0.01

The multivariate analysis (Table 5) demonstrated an independent influence of both age and comorbidities on incontinence, specifically diabetes on urinary bother (obstructive/irritative symptoms), both age and diabetes on sexual function/bother and both age and comorbidities on hormonal function (contrary effect). The patient groups ≤70 years and >70 years had an average age of 65 and 75 years, respectively. Thus, the hazard ratio corresponds on average to the symptom increase between a 65 year old and a 75 year old patient. A CCI>5 doubled the probability for individual urinary symptoms, erectile dysfunction or a great/moderate problem with urinary or sexual function overall (Table 6).

**Table 5:** Multivariate analysis considering the influence of age (>70 vs. ≤70 years) and comorbidities (1. any comorbidity, 2. specific comorbidities) on individual symptoms

question	risk factor	hazard ratio	95% confidence interval	p-value
leaking urine ≥once a day	comorbidity	2.2	1.2-3.8	< 0.01
	
	coronary heart disease	1.7	1.0-2.9	0.06
	
	diabetes	1.9	1.0-3.7	0.06

no complete urinary control	age	1.9	1.3-2.7	< 0.01

great/moderate problem with frequent urination	comorbidity	2.0	1.2-3.3	< 0.01
	
	diabetes	3.3	1.9-5.9	< 0.01

great/moderate problem with urinary function overall	comorbidity	2.2	1.2-3.9	< 0.01
	
	diabetes	2.5	1.3-4.8	< 0.01

erections not firm enough for intercourse	age	2.8	1.7-4.7	< 0.01
	
	comorbidity	1.7	1.0-2.9	0.04
	
	diabetes	7.0	2.3-21	< 0.01

great/moderate problem with sexual function overall	age	1.8	1.1-3.7	0.04
	
	comorbidity	1.9	1.1-3.5	0.03
	
	diabetes	2.9	1.3-6.2	< 0.01

hot flashes ≥once a day	comorbidity	3.5	1.0-12	0.05
	
	COPD	5.6	1.9-16	< 0.01

lack of energy ≥once a day	age	0.4	0.2-1.0	< 0.05
	
	comorbidity	4.2	1.4-12	0.01

great/moderate problem with hot flashes	COPD	4.5	1.5-13	< 0.01

great/moderate problem with lack of energy	comorbidity	2.7	1.1-6.4	0.03

COPD: chronic obstructive pulmonary disease

**Table 6:** Hazard ratios for individual symptoms with a Charlson Comorbidity Index of >5 vs. ≤5

question	hazard ratio	95% confidence interval	p-value
leaking urine ≥once a day	2.5	1.5-4.0	< 0.01

no complete urinary control	1.7	1.2-2.5	< 0.01

great/moderate problem with frequent urination	2.1	1.3-3.2	< 0.01

great/moderate problem with urinary function overall	2.1	1.3-3.2	< 0.01

erections not firm enough for intercourse	2.2	1.3-4.0	< 0.01

great/moderate problem with sexual function overall	2.2	1.2-3.8	< 0.01

COPD: chronic obstructive pulmonary disease

Comparing a particular group of prostate cancer patients who were scheduled for a permanent brachytherapy as monotherapy (n = 69) to the remaining patients (n = 459), a significantly younger patient age (mean 66 vs. 71 years; p < 0.01) and a smaller portion with comorbidities (48% vs. 64%; p < 0.01 - coronary heart disease in 10% vs. 24%: p < 0.01) became evident. HRQOL scores were significantly higher before treatment in the urinary, sexual and hormonal domains - with greatest mean differences (p < 0.01 for all) considering urinary continence function/bother (8 points respectively), obstructive/irritative bother/overall urinary bother (9 points respectively), and sexual function/bother (18 points respectively).

## Discussion

Patient age has a strong impact on HRQOL for prostate cancer patients before a primary curative treatment. Increasing urinary incontinence, urinary bother, and above all a strongly age-related decreasing sexual function has to be considered. Comparability of studies analyzing incontinence and impotence is often limited by different definitions. Urinary continence can be defined by wearing pads, dripping urine when coughing, any involuntary loss of urine or other definitions [23-25]. In the RTOG/EORTC (Radiation Therapy Oncology Group/European Organization for Research and Treatment of Cancer) grading system, urinary incontinence does not occur at all [26]. LENT SOMA (Late Effects Normal Tissues with Subjective, Objective, Management and Analytic categories) tables consider incontinence in some detail (e.g. grade 1 = < weekly episodes/occasional use of pads) [27]. HRQOL questionnaires allow an assessment with a higher accuracy from the patient's perspective.

Remarkable differences result between the percentage of patients who retain some ability to have an erection with increasing age and the percentage with erections firm enough for intercourse. Probably the best definition of potency (if only a single definition is used) is the ability to have an erection sufficient for penetration. This ability was found to decline particularly comparing the age groups ≤65, 66-70 and 71-75 years. Sexual function scores decreased remarkably between all age groups (mean decrease of 18 points between the age groups ≤65 and >75 years). Older patients accept a missing potency easier, so that changes of sexual bother scores were found to be less impressive (mean decrease of only 8 points between the age groups ≤65 and >75 years). Improving bother scores for patients with low scores before external beam radiotherapy have been shown in a recently published analysis [20], indicating some adaptation to the sexual problems.

Rectal domain scores have not been found to be age-dependent. However, some symptoms differed between the age groups. Older patients tend to have less frequent bowel movements - a consequence of declining metabolism and physical activity. Furthermore, the percentage of patients who observed bloody stools is decreasing. It is unclear, if bloody stools are actually not occurring or simply not noticed. An occasional bleeding rate of 12% in the patient group up to 65 years is remarkable. As rectal bleeding is considered an important sign of proctitis following radiotherapy, this high frequency stresses the importance of recording the presence of bleeding already before treatment. For example, a higher bleeding rate in a patient group with a lower dose to the rectum volume could only be explained by a higher bleeding rate already before treatment in a group of patients after post-prostatectomy radiotherapy [19]. Late rectal bleeding is a key dose-limiting end point in prostate radiotherapy with increasing incidence above a dose of 60 Gy [28]. In a study by Goldner et al. [29], 52 patients were reported with grade 2-3 EORTC/RTOG late rectal side effects - the reason was rectal bleeding for 50 patients (96%).

Hormonal function, as defined in the EPIC questionnaire, improved with higher patient ages. Testosterone levels are known to decline with age. Manifestations of testosterone deficiency include depression, irritability, weakness, diminished libido, reduced muscle and bone mass [30]. The results of this study suggest some adaptation to changing hormone levels. As both comorbidities and age (contrary effects on hormonal scores) are considered in the calculation of CCI, no effect of CCI differences was found on hormonal scores.

As recently reported by Bhojani et al. [31], select comorbidities have a very strong effect on urinary function and sexual function. In comparison to the younger patients (≤65 years), the incidence of comorbidities was higher with increasing age in our study. Comorbidities have an additional, independent impact on HRQOL. Focusing on specific comorbidities, hypertension - the most frequent comorbidity in this population - has not been found to influence any of the HRQOL domains. Diabetes mellitus, a disease known to ensue microvascular complications and neuropathy, had the strongest influence on urinary symptoms and, above all, sexuality. Both sexual function and sexual bother scores were affected with a mean decrease of 16 and 18 points, respectively. Diabetes is very well known as a predictor of late radiation morbidity [32,33]. In contrast to studies using only various toxicity grading scales [32,33], prospective HRQOL studies help to consider more accurately pre-existing symptoms. Coronary heart disease and independently, chronic obstructive pulmonary disease, were associated with decreased hormonal domain scores. These diagnoses are very well known to reduce normal physical activities. No correlation of comorbidities, similar to the patient age, was found with the rectal domain scores.

## Conclusion

Urinary continence and sexual function are the crucial HRQOL domains with age-related and independently comorbidity-related decreasing scores. Diabetes, not found to increase significantly with age in this population, was found to have the greatest impact on the urinary and above all on the sexual domain. The results need to be considered for the interpretation of studies comparing different primary treatments or longitudinal changes after a curative treatment.

## Competing interests

The authors declare that they have no competing interests.

## Authors' contributions

MP, KF, MJE have made substantial contributions to conception and design; MP and KF have made substantial contributions to acquisition of data; MP, KF, BG, MDP, BA, JK, HB, GJ, MJE to analysis and interpretation of data. MP has been involved in drafting the manuscript. KF, BG, MDP, BA, JK, HB, GJ, MJE revised it critically for important intellectual content. All authors have given final approval of the version to be published.

## Pre-publication history

The pre-publication history for this paper can be accessed here:

http://www.biomedcentral.com/1471-2407/9/296/prepub
